# Emotional dysregulation as trans-nosographic psychopathological dimension in adulthood: A systematic review

**DOI:** 10.3389/fpsyt.2022.900277

**Published:** 2022-08-29

**Authors:** Claudia Carmassi, Lorenzo Conti, Davide Gravina, Benedetta Nardi, Liliana Dell'Osso

**Affiliations:** Department of Clinical and Experimental Medicine, University of Pisa, Pisa, Italy

**Keywords:** emotional dysregulation, affective dysregulation, adults, ADHD, ASD, emotion dysregulation

## Abstract

**Introduction:**

Emotional dysregulation (ED) is characterized by inappropriate emotional reactions related to environmental or cognitive stimuli. In most recent years, increasing interest has been devoted to its definition and detection across mental disorders for its detrimental role progressively highlighted in both neurodevelopment and adult mental disorders, with implications on the severity of clinical manifestations. The aim of this systematic review was to evaluate and gather the scientific evidence about ED in adult psychiatric population to elucidate the concept of ED as trans-nosographic entity.

**Methods:**

The electronics databases PubMed, Scopus and Web of Science was reviewed to identify studies in accordance with the PRISMA guidelines; at the end of the selection process a total of 29 studies (*N* = 709; *N* = 658; *N* = 1,425) was included. All studies included assessed the presence of ED symptoms, by means of a validate scale in adult (>18 years of age), in clinically diagnosed patients as well as healthy control participants.

**Results:**

Our results suggest ED as a trans-diagnostic factor across multiple mental disorders, such as bipolar disorder, attention deficit hyperactivity disorder, autism spectrum disorder, personality disorders; a better definition of this concept could be helpful to interpret and clarify many clinical cases and improve their diagnostic and therapeutic management.

## Introduction

Over most recent years, the concept of Emotional Dysregulation (ED) has received increasing interest in psychiatry being generally used to describe altered regulation of emotional states both in children, adolescents, and adults. Today, despite scientific research has been increasingly focused on the study of ED its description is still debated.

Emotions are temporary qualitative states associated with sub-cortical (1), limbic (2), and cortico-frontal (3) processing in response to internal or external stimuli, that are defined as salient for each individual, capable of influencing physiological, somatic and behavioral responses by a cyclic mechanism of continuous feedback and progressive structural remodeling (4, 5). Despite the importance of “emotional problems” in clinical conditions has conducted to an intense search for causes, in which ED occupied a prominent place (6, 7), a shared definition of ED is still not yet available. In 1991, Thompson provided a definition of the Emotion Regulation (ER) as “a set of processes by which any individual assesses, inhibits, maintains, or modifies the intensity, frequency or duration of emotional reactions in order to have the appropriate social behavior or to achieve goals” (8). The concept of ED was introduced only years later, and as of today, one of the most welcomed definitions comes from Bunford et al. (9) as “an individual's inability to exercise any or all aspects of the modulatory processes involved in emotion regulation, to such a degree that the inability results in the individual functioning meaningfully below his or her baseline.” According to Shaw et al. (10), ED is composed of three main alterations: the first, is an inappropriate and excessive emotional reaction compared to social norms, secondly uncontrolled and rapid shifts in emotions and lastly an abnormal allocation of attention to emotional stimuli. As the concept of ED, in the general scientific overview, tends to focus mainly on negative emotions, ED is frequently defined by low frustration tolerance, irritability, susceptibility to negative emotional experience and emotional lability (11); that is in line with the new clinical category of “disruptive mood dysregulation disorder” introduced in the DSM-5 (12), created to describe severe and persistent irritability in children, manifested by intense and prolonged outbursts of anger and angry mood. Further, ED includes the inability to recognize and accept emotions alongside lacks in the ability to adaptively choose effective strategies to manage arising emotions (13, 14) also associated with an increased risk for various forms of psychopathology (15).

The alteration of the processes of emotion regulation belongs to a dimension that is increasingly depicted as trans-nosographic and which entails the need for new studies (16). The large spreading of ED symptoms in different kind of disorders, strictly supports the dimension concept of mental disorders as the result of multiple element interactions, which do not always lead to the overt diagnosis as described by textbooks, but rather define a spectrum of behaviors from normal to pathologic conditions.

The emotional dimension was initially the focus of the research into understanding the cytoarchitectural and functional properties of subcortical structures (17, 18), and for the evaluation of the effects induced by pharmacologically active substances (19, 20). Only later the emotional dimension was reconsidered as a consequence and as an index of a psychopathological outfit, which can be used for diagnosis and clinical classification. Therefore, the dysregulated emotional dimension turns out to be a clinical element, the result of a behavioral manifestation in response to a psychopathological condition of various kinds (genetics and environmental), that may occur across different mental disorders.

ED has been recently highlighted to share features in various childhood mental disorders including Bipolar Spectrum Disorders, Attention Deficit Hyperactivity Disorder (ADHD) and Autism Spectrum Disorder (21). Following the purpose of this paper, we will focus mainly on adulthood Bipolar Disorder (BD), ADHD, ASD and other mental disorders as psychopathological conditions presenting with an ED. Given these premises, our aim is to propose an objective examination of the studies based on scientific evidence that support the concept of ED as a trans-nosographic dimension in adults (22).

## Methods

### Literature search

A systematic search was conducted from 1 May to 3 October 2021 in accordance with the PRISMA 2020 guidelines (23) and using the electronic databases PubMed, Scopus and Web of Science. The following search terms, without filters, restriction or limits, were used to identify all potentially eligible records: (“emotional dysregulation” OR “affective dysregulation” OR “emotion dysregulation”) AND (“neurodevelopmental disorders” OR “autism” OR “ASD” OR “autism spectrum disorder” OR “attention deficit hyperactivity disorder” OR “ADHD” OR “schizophrenia spectrum disorders” OR “schizophrenia” OR “bipolar disorder” OR “post-traumatic stress” OR “post-traumatic stress disorder” OR “PTSD” OR “borderline personality disorder” OR “personality disorders”). All studies from 1st January 1976 to 3rd October 2021 were included in the databases search.

### Eligibility criteria

The criteria used to include studies in this review were as follows:

- Human studies.
- Studies that included only individuals of ages > 18.
- Study that used a validated scale to evaluate the ED.
- Articles available in English.

Because we aimed at investigating the ED in human subject, studies investigating ED in animal models, were excluded. Furthermore, studies in the form of review, case reports and editorials were also excluded.

### Screening and selection process

**Figure 1:**
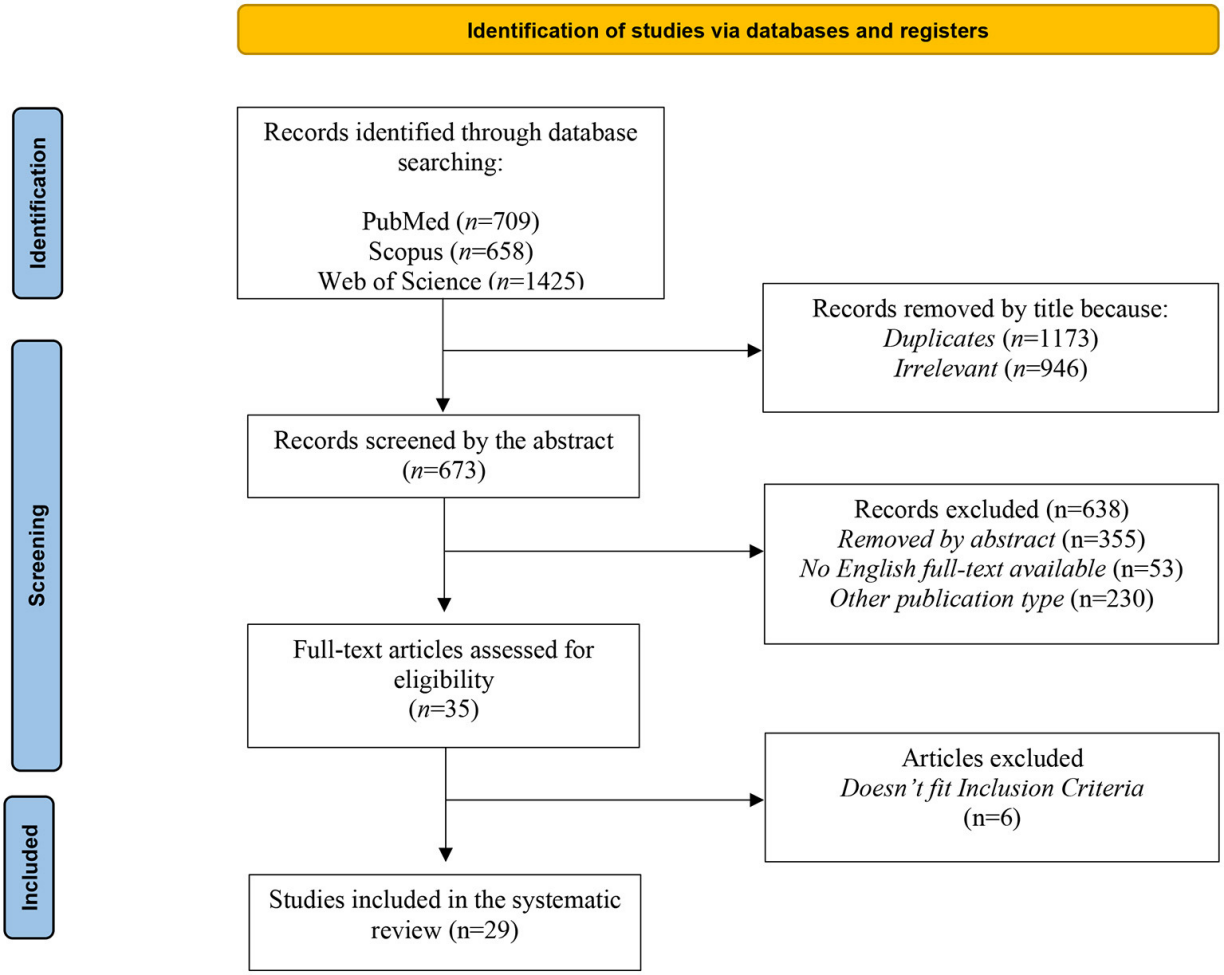
PRISMA flowchart of the study selection process. PRISMA, Preferred reporting items for systematic reviews and meta-analyses.

Three independent reviewers (L.C., D.G., B.N.) screened papers for inclusion and disagreements were resolved by discussion. The primary databases search produced a total of 2,792 records. After that, 2,119 articles were removed after titles because they were duplicates (*N* = 1,173) or not relevant (*N* = 946), and 638 were removed after abstract because not pertinent (*N* = 355), full text are not available or not in English (*N* = 53) or because they were other publication types (*N* = 230). After a full text reading other 6 articles were excluded because they didn't fit the eligibility criteria. Finally, a total of 29 articles were included in the present review. All three reviewers completed the process independently. We assessed the reference lists of selected papers for other eligible studies and any disagreement on included papers was resolved *via* discussion. The grade of agreement between the three authors was good. Decisions for inclusion or exclusion are summarized in a flowchart according to PRISMA 2020 recommendations (23). The study selection process is outlined in a flowchart (Figure 1).

### Quality assessment

The quality of articles included was assessed by a standardized tool adapted from Murad et al. (24). Furthermore, we used the Quality Assessment Tool for Observational Cohort and CrossSectional Studies (QATOCCSS) to asses the quality of the other type of study. Each study was scored as either “good,” “fair,” or “poor” (see Table 1). The quality assessment was performed by two independent reviewers (D.G. and L.C.) and a third reviewer (C.C.) cross-checked quality assessment results. Disagreements were discussed and resolved with the research team. The degree of agreement between the independent authors was good.

**Table 1:** Characteristics of the studies included in the systematic review.

**References**	**Country**	**Quality rating**	**Sample size**	**Population**	**Mean age**	**ED Scale**	**Psycho-pathological dimensions scale**	**Main findings**
Muñoz-Rivas et al. (35)	Spain	Good	120	VSR: *N* = 51; VSRSA: *N* = 49; VSRDA: *N* = 20	38.5	EPS-25	mSSI EAISR-SF	For VSR and VSRDA women, the Emotional Regulated group differs in post-traumatic symptoms
Newhill et al.	USA	Good	515	Schizophrenic patients: *N* = 90 Cluster B personality: *N* = 100 Student volunteers: *N* = 306 Healthy volunteers: *N* = 19	30.3 36.0 28.4	GEDM	//	Cluster B personality disorders patients showed higher GEDM scores
Faustino et al. (36)	Portugal	Fair	297	Psychiatric diseases: *N* = 60; HC: *N* = 231; Undiagnosed: *N* = 6	M = 32.7; F = 30.3	DERS; ERQ	QFC BSI	ED ↑ in clinical sample than HC
Ciuluvica et al. (37)	Italy	Good	283	CHR-DIS: *N* = 137; HEL-PER: *N* = 146	52.1	DERS	PANAS-TRAIT PANAS-STATE	The relationship between age and the study variables (D, ED, NA, NAT, NAS, PAT, PAS) in chronic diseases
Demir et al. (38)	Germany	Good	89	Syrian refugees resettled: Germany *N* = 49; Jordan *N* = 40	34.0	CERQ	PHQ-9 GAD-7 HTQ CTQ	Early life stress is positively correlated w/t maladaptive cognitive emotion regulation strategies and PTSD, anxiety severity and depressive symptoms
Anker et al. (39)	Norway	Fair	656	ADHD patiens: *N* = 656 (M = 351; F = 305)	43.5	CBS-SF	MINI ASRS	Lifetime AUD was not signicantly associated with ADHD or ED; Lifetime DUD was significantly associated with both hyperactivity-impuslivity and ED
Sàez-Suanes et al. (40)	Spain	Fair	121	ASD + ID: *N* = 121 (M = 81; F = 40)	35.5	ERC	DiBAS-R ASD-CA ASA-ASD-I GSD-LD CY-BOCS-PR DEX IUS-12-P	Maladaptive ER strategies were significantly detected in ASD+ID adults w/t greater anxiety symptoms
Pettorruso et al. (41)	USA	Good	428	NR = 151; LR = 160; HR = 69; PIU = 48	22.3	DERS	IADQ TPQ BIS-11 HAM	↑ DERS score significant positive correlation with increasing PIU risk-levels
Rogier et al. (42)	Italy	Good	180	GD patients: *N* = 79 (M = 64; F = 15); HC: *N* = 101 (M = 79; F = 22)	GD = 47.73; HC = 46.88	DERS; *ERQ*	PID-5 SOGS	↑ DERS positive predictor of SOGS score evaluating the severity of GD
Mestre-Bach et al. (43)	Spain	Good	98	GD patients: *N* = 98 (M = 89; F = 9)	42.7	DERS; ERQ	SOGS ASRS-v1.1	Direct association between ER and ADHD symptomatology/ GD severity
Rufenacht et al. (44)	Switzerland	Good	409	ADHD: *N* = 279; BPD: *N* = 70; BPD + ADHD: *N* = 60	35.5; 31.7; 30.3	ERS; CERQ; BES-A	ARSV-V1.1 BDI-II	ADHD patients had ↑ cognitive emotional regulation strategies and ↓ emotional reactivity than BPD patients.
Palagini et al. (30)	Italy	Good	77	BD w/t insomnia: *N* = 54; BD w/o insomnia: *N* = 23	47.6; 50.3	DERS	SCID-I BDI-II YMRS SSI	Insomnia symptoms resulted significantly correlated with ED, Emotional impulsivity and suicidality in BD subjects
Raudales et al. (45)	USA	Fair	209	Trauma-exposed patients: *N* = 209	37.9	DERS	SCID-V-RV PDS PCL-C PANAS-NA	Interpersonal trauma (especially sexual assault) in associated with ↑ ED
Liu et al. (46)	Singapore	Fair	150	SSD: *N* = 150	26.5	DERS CERQ	BCIS BPRS-E	Global ED was associated with more severe positive and depressive symptoms.
Bodalski et al. (47)	USA	Fair	159	ADHD: *N* = 59 Non-ADHD: *N* = 100	30.5	DERS ERQ	BAARS-8 AAQ-II CBAS BFIS NRI-RQV CES-D GAD-7	ADHD symptoms were associated in ↑ deficits in emotional regulation
Hirsch et al. (48)	Germany	Good	213	ADHD patients: *N* = 213 (M = 134; F = 79)	M = 33.5; F = 37.1	EMO CHECK	ASTM Qb+ AdultADHD-RS-sr CAARS-S CAARS-O BDI SCL-90-R GSI SCID-II	No significant differences between patients with or w/o comorbidity
Garofalo et al. (49)	Italy	Good	399	HC *N* = 399 (M = 226; F = 173)	37.9	DERS	MCMI-III BIS-11	ED and DERS scores significantly and positively related to schizoid, schizotypal, avoidant, antisocial and borderline PD traits; but significantly and negatively related to histrionic, narcissistic and obsessive-compulsive PD traits
Rufino et al. (50)	USA	Good	156	BPD *N* = 156; M = 60; F = 96	29.4	DERS	C-SSRS WHODAS 2.0 SCID-I/-II	BD with Global Dysregulation subjects reported significantly ↑ functional impairment and suicidal ideation than other groups
Terzi et al. (51)	Italy	Fair	79	BPD *N* = 79 (F = 63; M = 16)	34.0	DERS	BIS-11 A-Q SHI	DERS score significantly account for aggressive proneness and for self-harm in BPD sample
Corbisiero et al. (52)	Switzerland	Fair	514	Adult ADHD: *N* = 393; Childhood ADHD: *N* = 68	32.3	ED	WRAADDS CAARS WURS-k AI	↑ ED is major indicator of the severity of ADHD independently of a comorbidity disorder
Lagerberg et al. (53)	France	Poor	372	BD patients: France sample *N* = 329; Norway sample *N* = 43	41.7; 32.1	ALS-SF	DIGS SCID-I YMRS MADRS IDS-C	ALS-SF scores showed how the presence of a lifetime AUD was significantly associated with ↑ affective lability in BD
Chamberlain et al. (54)	USA	Fair	423	ADHD symptoms: *N* = 86; HC: *N* = 337	22.3	DERS; BIS-11	ASRS SCI-GD QOLI RSE CANTAB	Compared to HC, the ADHD symptom group showed significantly ↑ emotional dysregulation and impulse control disorders, and ↓ lower quality of life and self-esteem
Richard-Lepouriel et al. (55)	Switzerland	Good	533	ADHD: *N* = 150 (M = 99; F = 51); BD: *N* = 335 (M = 151; F = 184); HC: *N* = 48 (M = 23; F = 25)	GD = 37.7; BD = 44.0; HC = 39.5	ALS; AIM	ASRS WURS DIVA 2.0 DIGS	EL and ER ↑ in ADHD subjects than BD; Axis-1 comorbidities ↑ ALS and AIM scores exc BD
Ruscitti et al. (56)	USA	Fair	404	ED patiens: (EDNOS: *N* = 120; AN: *N* = 29; BN: *N* = 22; BED: *N* = 20) No-Ed patients: (DD: *N* = 115; AD: *N* = 131; SUD: *N* = 117	31.2	DERS	SCID-I	To improve ER skills training for patients with EDs, particularly those with BED and EDNOS
Bayes et al. (57)	Australia	Good	190	BP *N* = 83; BDP *N* = 54; BDP+BP *N* = 53	35.4; 32.9; 36.2	DERS; CERQ	//	Adaptive emotion regulation strategies were ↑ in the bipolar group compared to both BPD and comorbid groups
Yoon et al. (58)	USA	Poor	101	N: 101 (M = 46; F = 55)		DES	PPI-R	Association between psychopathic traits and emotional regulation.
Scott et al. (59)	USA	Poor	100	BPD: *N* = 100 (M = 35; F = 65)	45.9	EERI; ATQ; ERQ; DERS	SIDP-IV NEO-PI-R CTS2	Emotional Regulation was strongly related to BPD than ASPD and AVPD
Beblo et al. (60)	Germany	Fair	39	BPD: *N* = 19; HC: *N* = 20	29.0; 31.9	ERQ; DERS	SCID-I/-II Mini-DIPS MSCEIT TEMINT LPS-K	BPD subjects had ↓ ER strategies, impulse inhibition and emotional awareness, but no Emotional Intelligence Impairment than HC
Newhill et al. (61)	USA	Fair	100	WM: *N* = 9; NWM: *N* = 10; NWF: *N* = 27; WF: *N* = 53%	36	EDM AIM	PANAS TAS IIP-PD	The GEDM demonstrates good reliability and validity and correlates significantly with other established measures of affect.

VSR, Victims with a Single Report; VSRSA, Victims with Several Reports by the Same Aggressor; VSRDA, Victims with Several Reports by Different Aggressors; ADHD, Attention Deficit Hyperactivity Disorder; PTSD, Post-Traumatic Stress Disorder; AUD, Alcohol Use Disorder; DUD, Drug Use Disorder; ASD, Autism Spectrum Disorder; ID, Intellectual Disabilities; NR, No Risk; HR, High Risk; PIU, Problematic Internet use; SOGS, South Oaks Gambling Screen; GD, Gambling Disorder; BPD, Borderline Personality Disorder; BD, Bipolar Disorder; PD, Personality Disorder; HC, Healthy control; SSD, Schizophrenia Spectrum Disorders. CHR-DIS, Chronic disease; HEL-PER, Help personality; EDNOS, Eating disorder no other spec; DD, Depressive disorder; AD, Anxious disorder; SUD, Substance use disorder.

## Results

A total of 29 publications were provided by the search, any of them are studies ranging from 2004 to 2021. Details of each study included in the review are reported in Table 1.

### Characteristics of the study samples

#### Population

In the present search, 24.13% of the studies included ADHD patients (*n* = 7), 17.24% of the samples included patients with BD (*n* = 5) and another 17.24% included Borderline Personality Disorder patients (*n* = 5), with all the studies including both genders. Of the remaining studies, 6.89% included patients with Gambling Disorder (*n* = 2), 3.44% ASD (*n* = 1), 3.44% Problematic Internet Use (*n* = 1); 27.58% of the remaining included non-clinical (*n* = 4).

### Emotional dysregulation assessment

To assess ED, 17 studies (58.62%) used one scale only. The most utilized scale was the Difficulties in Emotion Regulation Scale (DERS) (*n* = 17; 58.62%), 9 studies utilized this scale in combination with another one, six alongside of the Emotion Regulation Questionnaire (ERQ), two with the Cognitive Emotion Regulation Questionnaire (CERQ), one with the Emotional Experiencing and Regulation Interview (EERI), the Adult Temperament Questionnaire (ATQ) and the ERQ, and one with the Barratt Impulsiveness Scale (BIS-11). Four studies used the CERQ (13.79%) of which one alone, two alongside the DERS and one with the Emotion Reactivity Scale (ERS) and the Base Empathy Scale in Adults (BES-A). Six studies used the ERQ, (20.68%), all in combination with the DERS. Two studies utilized the Affective Liability Scale (ALS) (6.89%), one in combination with AIM and one using both the extended and the shortened version; one study used the ED scale only (3.44%); one used the EMO CHECK Battery (3.44%); one the Current Behavior Scale Self Report (CBS-SF) (3.44%); one the Emotional Processing Scale (EPS-25) (3.44%); one the Emotion Regulation Checklist (ERC) (3.44%); one the Differential Emotion Scale (DES) (3.44%); one the General Emotional Dysregulation Measure (GEDM) (3.44%), and one the Emotional Dysregulation Measure (EDM) (3.44%).

### Emotional dysregulation across mental disorders

#### Bipolar disorder

The emotional dimension may play a relevant role in various life phases of patients suffering from Bipolar Disorder (BD). Patients with BD exhibit ED when compared to healthy controls, and display a greater overall difficulty regulating emotions (25), even during remission from mood episodes (26, 27); they clearly show a tendency to use negative strategies to regulate mood, such as rumination and catastrophizing (27). The depressive phase also involves a picture of dysregulation but is characterized by a marked inhibition of positive emotions and a predilection for negative attitudes and emotions, among which anhedonia and affective flattening dominate. In bipolar disorder type II, the dysregulated dimension of emotions in the manic component is less pronounced and tends to be hidden in the euthymic phases of the rest of the population (28). A small number of studies have focused on the comparison of the specific BD subtypes: for instance, Van Rheenen et al. (27) reported the lack of differences in ER between bipolar I and II groups, while Fletcher et al. (29) reported that BP II (but not BP I) patients were more likely to engage in emotion-focused and self-focused rumination about positive affect, compared to unipolar depression participants (29). A particularly important element is the assessment of the sleep pattern in patients with BD. In a sample of patients with BD type II diagnosis, the sleep pattern alteration determines an emotional alteration that leads to an increase in impulsiveness levels, resulting in a substantial and statistically relevant increase in suicidal behavior. Therefore, a careful assessment and an early intervention on sleep and emotional dimension could have a significant positive impact on the prognosis of the disorder (30). Various empirical studies thus establish and acknowledge that BD is a disorder of emotion and motivation. In particular, the orbitofrontal cortex and the amygdala are involved and the connection between these two regions is a marker of biological vulnerability in mood disorders (31). Therefore, ED was proposed to be associated not only with negative functional outcomes in BD patients (27) but has also been proposed as a central component in the development and maintenance of mood disorders and reinforcement of mood instability as well as being correlated to impulsive behaviors, and increased risk of suicidality in individuals (32–34).

In this contest, it is interesting to notice how different studies have identified ED as a nuclear factor of the Cyclothymic Disorder, is characterized by a chronic manifestation of low-grade depressive and hypomanic symptoms (12). Recent studies have highlighted how the Cyclothymic Disorder and the Cyclothymic Temperament are characterized by an elevated level of emotional and behavioral instability and over-reactivity (62, 63). Furthermore, the cyclothymic temperament was shown to be associated with an increased mood and emotional reactivity, with intense reactions to external stimuli, great irritability, anxiety and scarce impulse control (62).

#### Autism spectrum disorder

ASD is a neurodevelopmental disorder, characterized by social-communication difficulties, the presence of repetitive behaviors and/or restricted interests with alterations in the sensory profile (12). Children with ASD are at risk of developing internalizing and externalizing problems, moreover both of them are recognized as important predictors of the development of the child's social competence. Dysregulated “externalizing” behaviors are represented by aggressiveness, impulsivity, and control problems, while dysregulated “internalizing” behaviors are depicted by withdrawal, anxiety, and depression (64–66).

ED has been confirmed as a trans-diagnostic risk factor for multiple disorders or symptoms, especially the ones concerning internalizing problems (15, 67). Given that those kinds of problems are a major pillar of ASD, and that multiple studies suggested that emotional regulation is impaired in ASD (67, 68), deficits in emotional regulation may help explaining increased rates of comorbid disorders in ASD patients (68, 69). A study carried by Sàez-Suanes et al. (40) reported that adults with ASD with greater anxiety symptoms also showed greater use of maladaptive emotional regulation strategies, therefore people with ASD are more vulnerable to manifest anxiety for their difficulties in managing negative emotions. In a more global view, some models suggest that anxiety in the general population could be based on emotional regulation and intolerance to uncertainty (70–72). Furthermore, affected individuals are particularly exposed to altered inhibition of emotional response (73), given the recognized difficulties they have in reading situations, identifying with others, assessing perspectives (74), leading to angry and excessive behavioral outcomes such as breaking down, hitting others, screaming with real fits of rage, the previously called “externalizing” behaviors.

Following the lead, many authors have suggested that ED may be an effective explanation to define the typical manifestations of externalizing behaviors (such as anger and aggression) and internalizing behaviors (like anxiety and depression) (15, 75, 76), specifically in adult patients with ASD, defining an extremely typical trajectory of the disorder (68, 74, 77–79).

Gender differences are also reported for ED in ASD patients. In an analytic sample, females have significantly higher emotional dysregulation than males when appropriately investigated (80). Given the gender disparity in ASD diagnosis and given potential differences in the manifestation of autistic traits in females compared to males (81–84) investigating emotional elements in advance could play an index factor in the correct diagnosis of ASD in females, given the male trend in diagnosis, proposing a trajectory and diagnosis perspective that in the most decisive cases of ED leads to complex and organized cluster B personality disorders as hypothesized in an adult trajectory of subthreshold autistic conditions.

#### Attention deficit hyperactivity disorder

ADHD is a neurodevelopmental disorder that persists into adulthood in ~50–60% of cases (85, 86) with a prevalence of 2.5–4.4% (87–89). It's characterized by inattention, impulsivity, and hyperactivity but also problems with mood instability, such as irritability, swift mood changes, hot temper, and low frustration tolerance, frequently appear to accompany the disorder (90). ADHD is a disorder in close relationship with ED (10, 91), in children, adolescents and adults (92–94) and it is estimated that 37–40% of individuals with ADHD have ED (95). Individuals with ADHD and ED are more impaired in daily life functioning than those with ADHD in the absence of ED (9, 47, 96–100).

A growing number of evidence are showing that ED symptoms are expressed in a large percentage of adult ADHD patients (99, 101–103), in particular in one adult study, 55% of ADHD patients showed ED that was of greater severity than >95% of control subjects (98, 104) emphasizing the importance of screening the emotional domain of ADHD. A first step was made by Wender (105), who described symptoms of ED in the Utah Criteria for the diagnosis of ADHD in adults and, later on, many other authors took into account the emotional experience of ADHD patients and proposed the consideration of emotional symptoms as an additional core component of ADHD psychopathology (106–108). However, in the last revision of the DSM-5-TR and ICD, ED is still not included as a third central dimension in the psychopathological core of the ADHD picture, although it's recognized its importance and its use is recommended for optimal framing (109). The dimension of ED is highly non-specific due to its trans-nosographic nature, the lack of standardization and, above all, the extreme subjectivity of behavioral manifestation that varies from individual to individual.

Various studies have shown that individuals with mental disorders have a tendency to use fewer effective strategies for emotion regulation, and particularly in ADHD it seems that the difficulty is related to the temporal dimension and the predilection for short-term rewards (16).

The severity of ADHD symptoms, as argued in the meta-analysis study conducted by Beheshti et al. (110), is significantly correlated with ED dimensions such as emotional lability and emotion recognition.

Although being correlated with all the core domains of ADHD (102, 111, 112), ED appears to be part of the same dimension of the hyperactivity/impulsivity symptoms, with which it shares a much stronger relationship compared to the attention deficit core (74, 101, 113, 114). Therefore, the classic domains of inattention, hyperactivity and impulsivity are not sufficient to explain the entire spectrum of symptoms in adults, especially when the disorder is found with other comorbid pathologies, so the assessment of the emotional dimension is fundamental for a better diagnostic framing and to avoid evaluating ED as a secondary effect due, for example, to an anxiety-type framework (52, 115).

#### Schizophrenia spectrum disorders

Schizophrenia Spectrum Disorders are severe psychiatric conditions characterized by a varying severity of positive and negative symptoms. Patients with symptoms of severe ED have structural alterations in the limbic system, in particular a lower density of gray matter in the right ventral striatum has been shown (116).

A previous review and meta-analyses of the available literature has reported that patients with schizophrenia are likely to use more maladaptive and less adaptive cognitive emotion regulation strategies compared to healthy controls (117) and that they are usually related to the maintenance and exacerbation of positive symptoms (118, 119). Maladaptive cognitive emotion regulation strategies were also found to increase the tendency for cognitive biases and misinterpretations of negative affective states, and return increase the risk of hallucinations and delusions (120). Patients with schizophrenia spectrum disorders are known to experience greater emotional distress (121). Some patients may engage in expressive suppression to cope with painful emotions. However, habitual use of expressive suppression is likely to increase arousal, amplify distress and worsen the course of positive symptoms (119).

In 1997 Gross and Levenson suggested that there may be important differences between the ability to regulate positive and negative emotions and later many other studies focused on the importance of ED in the psychopathology of schizophrenia. A study from Owens et al. (122) highlighted a positive correlation between global ED and both positive and negative symptoms; another from Lincoln et al. (123) observed that global ED and its components (e.g., emotional awareness, tolerance, acceptance, and modification) were negatively correlated with paranoia symptoms; contrasting results came instead in a study carried by Bonfils et al. (124) were they did not observe any significant correlations between global ED and positive or negative symptoms.

It is therefore important for future research to extend the current findings by examining the ability of schizophrenic patients to regulate these two entities, especially the processes of worry and anxiety may be causal elements of delusional symptoms, so a thorough investigation and assessment of the emotional dimension and application of treatment could result in an improved prognosis of schizophrenic disorder (125).

#### Post-traumatic stress disorder

ED has been recently considered a fundamental component of the Post-traumatic Stress Disorder (PTSD) as the global literature on emotional regulation and intensity of PTSD symptoms suggests there is a general lack of emotional regulation in people exposed to various traumatic events (35). A recent meta-analysis reported that PTSD, regardless of the kind of traumatic event that generates it, is characterized by a general dysregulation of emotions that is more relevant in explaining the symptoms than any of the specific regulatory strategies used individually (126). In addition, ED gives rise to the hyper-vigilance and distortions of attention, hyper-excitement, emotional numbness and irritability (127). In support to the hypothesis, from a neuroanatomical perspective, the amygdala and insula are particularly hyperactive in response to stimuli that convey social threats (e.g., faces), aversive images, and signals that evoke personal trauma, in patients with PTSD.

The latest literature is greatly supporting the association between PTSD and constructs of emotional value, like: suppression (intended as the inhibition of expression of emotional responses (128), experiential avoidance (the attempt to suppress unwanted internal experiences such as emotions, thoughts, or memories (129), alexithymia (difficulty identifying what they feel (130), or dissociation (the mental process of disconnection from thoughts, and feelings (14). Litz et al. (131) defined the concept of “Emotional numbness” with the disaffection for emotion-provoking circumstances and the a lack of emotional reactivity, and furthermore proved it to be a relevant predictor of the endurance of post-traumatic symptoms. Besides, the 2014 study conducted by Abigail further defines the centrality of ED within PTSD and argues how it's the framework of ED that determines the presence of dissociative symptoms in PTSD. As PTSD can have a devastating impact on health and functioning, increasing the risk of comorbidity with other mental disorders (132) and adverse medical conditions [e.g., (133, 134)], therefore it is crucial to identify factors that can increase its risk early and preventively. These include ED, reflecting deficits in an individual's ability to recognize and accept their emotions, as well as deficits in the ability to adaptively choose effective strategies to manage emotions when they arise (13, 14). In fact, a great number of studies, suggest that the use of maladaptive strategies (i.e., self-blame, rumination, catastrophizing) may increase the vulnerability toward psychopathology, whereas adaptive cognitive styles (i.e., acceptance or positive refocusing and putting into perspective) can lead to more resilience to symptoms of psychological distress (135, 136). Researches showed that trauma, especially enduring or repeated traumatic experiences such as early life stress, seems to compromise the acquisition of appropriate emotion regulation skills (38). On that way, data indicate that the assessment of ED in standard medical care would allow the identification of those who are at risk of developing severe forms of PTSD (as they already possess a dysregulated emotional framework) providing a practical treatment target (132, 133, 137), given the association of ED with a variety of mental disorders.

#### Borderline personality disorder

Borderline Personality Disorder (BPD), is characterized by a pervasive pattern of instability of interpersonal relationships, self-image and affects, marked impulsivity, emotional instability, including impaired emotional awareness and “clarity” (138), problems employing emotion regulation strategies (139, 140), interpersonal difficulties and dysfunctional cognitive processes (141), with all of the above influencing the patients general functioning (142), treatment compliance (143, 144), physical health status (145) and relationship dimension (146).

Since the regulation of emotions is difficult in these patients, BPD perfectly fits in our examination, and ED should be central in clinical observation. Impulsivity and ED are pivotal and significant features in BPD over time (147). As argued in Sebastian et al. (148), the impulsivity seen in BPD (if not caused by ADHD) may be just another dependent and secondary element of ED; inter alia impulsivity is strongly associated with suicide attempts in both adults and adolescents (149–151) and non-suicidal self–injury (152, 153), whether it is planned or unplanned (154) meanwhile ED has been found to be the strongest predictor of self-harm over time (155–158) and serves as a maladaptive strategy to reduce negative affect and to regulate the mood (159, 160). Older patients diagnosed with BPD show alterations in emotion social functioning and regulation strategies, using maladaptive cognitive strategies such as rumination (161, 162) and thought suppression (163, 164); in contrast, younger people diagnosed with BPD are more likely to express anger and self-injurious behavior.

Other studies have instead focused on the role of ED in determining aggressive behavior (165–167) and behavioral dyscontrol (51, 168) in BPD.

Despite being the most extensively studied personality disorder (169, 170), its diagnosis is still quite problematic. BPD can be conceptualized as a severe mental disorder that continues over time with changing manifestations. Age-related symptoms should be considered in the diagnosis of BPD with the need for appropriate treatment focusing on ED and impulsive behavior (171). Focusing on the regulation of individual emotions in couple interaction, it seems to be a promising target to reduce relationship dysfunctions in BPD. This element contributes to enrich the mosaic of information that tends to centralize and consider as a dimension of necessary importance ED (50, 172, 173). As argued in the study conducted by Peter et al. (174), the application of practices of teaching or managing and understanding emotions by the therapist to the BPD patient, contributes to a substantial improvement in the psychopathological picture. In this setting, the use and implementation of a specific treatment practice such as dialectical behavior therapy (DBT) has been described. DBT mainly intervenes in the emotional dimension (175).

#### Other mental disorders

Regarding disruptive behavior disorders—impulse control and conduct disorders, nutrition and eating disorders, substance-related and addiction disorders, paraphilic disorders—the ED dimension is particularly evident. Many studies have highlighted the presence of ED in many mental disorders: oppositional defiant disorders (111, 115), borderline personality disorders (176, 177), mood disorders, (178, 179), anxiety disorders (33), eating disorders (180), and substance abuse/dependency (181).

By analyzing each psychopathological nucleus, the perspective of ED assessment is intended to challenge the current clinical-diagnostic dimension. In fact, emotional phenomena are rarely evaluated as primary dimensions of alterative processes, and more often as a derived and derivable dimension due to environmental effects, maladaptive and secondary to other psychiatric pathologies.

## Discussion

The aim of this review is to bring an essential vision of the concept of ED and its role in the frame of the adult psychopathology and its relevance in the clinical practice. Where the importance of ED is prominent in the study of children and adolescents' mental disorders, not many studies have yet focused on its role in the development and maintenance of adult mental disorders.

In the recent years, ED has been a new focus of interest in the adult psychiatric research, although to this day it still lacks of a univocal definition. One of the most commonly accepted, and the one we choose to relate to, pictures ED as the “inability to exercise any or all aspects of the modulatory processes involved in emotion regulation, to such a degree that the inability results in the individual functioning meaningfully below his or her baseline” (9). To this day, the most widely used method of assessment of ED, is the DERS scale, both developed and evaluated in an adult sample (182). It's a self-report questionnaire, structured in six domains exploring Awareness (acceptance of emotions), Clarity (knowledge about one's emotions), Goals (evaluating difficulty with goal-directed behavior when upset), Impulse (indicative of difficulty with behavioral control when experiencing negative emotions), Non-acceptance (indicative of negative secondary emotions) and Strategies (the belief that no strategy can decrease negative emotion) (97).

Researches in literature proves that in many mental disorders, such as ADHD, psychosis, or affective mood disorders, ED is an essential but often neglected part of psychopathology. We aimed at assessing ED as a trans-nosographic entity across the main mental disorders, highlighting its role in the development, manifestation and maintenance of such pathologies.

In many disorders, such as BD, PTSD, and Schizophrenia, ED has a major role in the development and maintenance of the symptomatology; in BD acts as a reinforcement of mood instability and is associated to impulsive behaviors, and increased risk of suicidality (32–34). Also studies in BPD patients have confirmed ED as the strongest predictor of impulsivity and self-harm over time (148, 155–158). Both BD and Schizophrenia, patiens have also shown a greater difficulty regulating emotions during episodes as well add during remission (25–27, 117) and in Schizophrenia being related to both positive and negative symptoms (118, 119, 122). In neurodevelopement disorder such as ASD and ADHD, ED represent an explanation for most of the externalizing and internalizing behaviors (15, 75, 76), specifically in adults. Evermore, a growing number of studies are confirming the close relationship between ADHD and ED and how the latter is strongly correlated with all the core domains of ADHD (102, 111, 112). Even PTSD, regardless of the kind of traumatic event that generates it, is knowingly characterized by a general dysregulation of emotions (126), giving rise to hyper-vigilance, hyper-excitement, emotional numbness and irritability (127).

ED has thus confirmed it pivotal role as trans-diagnostic risk factor for multiple disorders and symptoms, especially the ones concerning internalizing problems (15, 67). In this view, it is strongly highlighted both the importance of conducting an attentive assessment of the strategies of emotional regulation during the diagnostical framing of clinical practice and the therapeutic central role that it occupy.

## Conclusions

The inclusion of ED as a trans-nosographic entity could be stimulant or provocatory, but would perfectly fit the vision of mental disorders as a wide range of behavioral conditions that progressively move away from conventional, through the most intrinsic dimensions of each individual and how they tend to relate to the external environment. ED and related symptoms are well integrated in this kind of clinical features and could be a helpful concept to interpret and clarify many clinical cases.

The concept of ED will probably help in discovering basic approaches to the understanding, diagnosis, and treatment of mental disorders. ED may possibly be a new concept, allowing mental disorders to be defined by their essential nature instead being defined by their phenotype and objectively measured symptoms.

## Limitations

When discussing our results some limitations should be taken into account.

Firstly, the dimension of ED is highly non-specific due to its trans-nosographic nature, the lack of a univocal definition, especially when investigated in adulthood, the variety and heterogeneity of the scales available for its assessment and, above all, the extreme subjectivity of behavioral manifestation that varies from individual to individual. Second, studies on ED are mainly centered on the pediatric population, therefore data on its manifestation and its association with some mental disorders in adulthood, are still scant. However, one of the aims of the present review was to shed light on the need for further studies in adult population. Third, due to the heterogeneity of ED nomenclatures, the terms we chose to include in the literature search might not be comprehensive of all the terminology used to this day. Lastly, during the selection process we didn't use controlled vocabulary (e.g., MeSH) and we included only articles in English language.

## Data availability statement

The original contributions presented in the study are included in the article/supplementary material, further inquiries can be directed to the corresponding author/s.

## Author contributions

CC: conceptualization, methodology, investigation, writing—original draft, writing—review and editing, and supervision. LC and DG: methodology, investigation, writing—original draft, and writing—review and editing. BN: methodology, investigation, writing original draft, and writing—review. LD'O: supervision and final manuscript version revision. All authors contributed to the article and approved.

## Conflict of interest

The authors declare that the research was conducted in the absence of any commercial or financial relationships that could be construed as a potential conflict of interest. The reviewer MB declared a shared affiliation with the authors to the handling editor at the time of review.

## Publisher's note

All claims expressed in this article are solely those of the authors and do not necessarily represent those of their affiliated organizations, or those of the publisher, the editors and the reviewers. Any product that may be evaluated in this article, or claim that may be made by its manufacturer, is not guaranteed or endorsed by the publisher.
